# Quantitative analysis of chest computed tomography in alpha-1-antitrypsin deficiency

**DOI:** 10.1186/s12931-025-03413-4

**Published:** 2025-11-15

**Authors:** Philipp Höger, Johanna Benholz, Oliver Weinheimer, Mark Oliver Wielpütz, Claus Peter Heußel, Arved Bischoff, Ralf Eberhardt, Katharina Buschulte, Sebastian Fähndrich, Julia D. Michels-Zetsche, Hilal Ersöz, Arturo Olivares Rivera, Felix Herth, Franziska C. Trudzinski

**Affiliations:** 1 Department of Pneumology and Critical Care Medicine, German Center for Lung Research (DZL), University of Heidelberg, Translational Lung Research Center (TLRC-H), Heidelberg, Germany; 2 Department of Diagnostic and Interventional Radiology, German Center for Lung Research (DZL), University Hospital Heidelberg, Heidelberg, Germany, Translational Lung Research Center Heidelberg (TLRC), Heidelberg, Germany; 3 Department of Diagnostic Radiology and Neuroradiology, University of Greifswald, Greifswald, Germany; 4 Department of Diagnostic and Interventional Radiology with Nuclear Medicine, Thoraxklinik at University Hospital Heidelberg, Heidelberg, Germany; 5 Department of Pneumology and Critical Care Medicine , Asklepios Klinik Barmbek, Hamburg, Germany; 6 Department of Pulmonology, University Medical Center Freiburg, Medical Faculty, Freiburg, Germany; 7 Department of Pneumology and Critical Care Medicine, Thoraxklinik University of Heidelberg, Röntgenstraße 1, Heidelberg, 69126 Germany

**Keywords:** Alpha1-antitrypsin, Alpha1-antitrypsin deficiency, Chronic obstructive pulmonary disease, Chest computed tomography, QCT

## Abstract

**Background:**

Chest quantitative computed tomography (QCT) has been used in clinical trials to monitor patients with alpha-1 antitrypsin deficiency (AATD). There is limited data on the use of quantitative computed tomography (QCT) to phenotype emphysema in AATD.

**Methods:**

Data from patients with a reduced AATD serum level and/or at least one deficiency mutation, who underwent a chest CT scan at the Thoraxklinik, University of Heidelberg, between 03/2012 and 02/2024, were retrospectively analyzed. The patients were categorized into three groups based on their AAT serum levels: reduced to normal (> 70 mg/dl), moderate (41–70 mg/dl), and severe (≤ 40 mg/dl). The QCT analyses were performed using a fully automated quantitative CT software package (YACTA v2.9.4.98).

**Results:**

In this retrospective cohort study, 75 AATD patients were analysed, including 13 with reduced-to-normal AATD, 16 with moderate AATD and 46 with severe AATD. The mean age was 54.3 ± 14.7 years with no differences between groups. Significant differences in pack-years (PY) were found: reduced to normal 14.9 ± 17.2, moderate 39.4 ± 31.9 and severe 15.1 ± 14.6 (*p* < 0.001). There were no differences in predicted FEV1% (54.1 ± 28.9%). QCT emphysema parameters showed a decrease in mean lung density and 15th percentile with lower serum levels (*p* < 0.05 for each), but no differences in global emphysema index (EI). A lobar-specific analysis, revealed that the reduced-to-normal and moderate AATD groups exhibited upper-lobe predominant emphysema. In contrast, the severe AATD group showed basal-predominant emphysema with maxima in the middle lobe and lingula. Additionally, a binary logistic regression analysis identified age (CI 1.01–1.12, *p* = 0,024) and lower of AATD serum levels (CI 0.92–0.98, p = < 0.001) as significant indicators for an emphysema pattern that was predominantly localized in the middle lobe and lingula.

**Conclusion:**

The study provides new insights into the distribution of emphysema, with lobar-specific quantitative analysis challenging the existing paradigm that emphysema in severe AATD is most pronounced in the lower lobes.

## Introduction

 The main physiological function of alpha-1-antitrypsin (AAT) is to protect lung tissue from enzymatic degradation, particularly by inhibiting neutrophil elastase. Consequently, alpha-1-antitrypsin deficiency (AATD) leads to the early onset of emphysema due to increased proteolytic activity [1]. AATD is an autosomal codominantly inherited disease. The risk of developing emphysema is significantly increased in homozygous and complex heterozygous carriers, such as the most common genotype for severe AATD Pi ZZ, while the risk is only slightly increased in heterozygous carriers, such as the Pi MZ genotype, or in genotypes associated with a moderate reduction in serum AATD levels, such as the Pi SZ genotype [1]. Studies and clinical registries have shown that, in addition to the genotype, environmental factors and smoking have a major influence on the development of emphysema in AATD, and the clinical course of people with AATD is very heterogeneous and often difficult to predict. By age 40, only 5–8% of non-smokers with severe AATD deficiency develop emphysema, whereas the prevalence in smokers is 67% [2]. Computed tomography (CT) lung imaging is used as a non-invasive method to assess changes in lung structure. CT can quantify the extent of emphysema through examiner-dependent visual analysis and automated procedures, i.e. quantitative CT (QCT) [3]. QCT biomarkers can be used in COPD patients to differentiate between phenotypes with the typical clinical features of emphysema-dominant and airway-dominant disease [4], to map the severity of the disease and also provide indications of possible disease progression [3]. QCT biomarkers have also been shown to be useful in AATD. The best example is PD15 as a marker of lung density, which was used as the primary endpoint in the largest randomised, placebo-controlled study to date on the efficacy of augmentation therapy in patients with AATD (RAPID) [5]. In addition, quantitative CT has been shown to be superior to the determination of lung function parameters, especially FEV1, for predicting all-cause mortality in patients with severe AATD [6]. Overall, QCT is used in patients with AATD, particularly for monitoring and follow-up of pulmonary emphysema, but little information is available on QCT markers in different phenotypes.

The aim of our analysis was to investigate pulmonary emphysema in patients with different AAT serum levels and genotypes in depth using QCT.

## Methods

### Study patients

This is a retrospective analysis. All patients with AATD who presented to the Thoraxklinik Heidelberg between 03/2012 and 02/2024 and underwent a computed tomography of the thorax were included. The study was approved by the ethics committee of Heidelberg University Hospital (S-S-495/2023).

AATD was either diagnosed at the AAT Centre at the Thoraxklinik Heidelberg or patients were referred from external practices after being diagnosed with AATD. As part of the diagnostic process, the serum levels of AAT were measured in all patients during the infection-free interval and stable disease state. Concurrently, the laboratory determined the levels of C-reactive protein. Genotyping was carried out in the AAT laboratory of the University Hospital, UKGM Marburg, for those patients who had not previously received such a measurement; if external results were available, these were requested. In the case of contradictory results between polymerase chain reaction genotyping and isoelectric focusing (IEF) or serum levels, or if there was evidence of a rare mutation, the seven exons of the SERPINA1 gene were analysed by next-generation sequencing (Progenika Biopharma, S.A.). Three patients were diagnosed with moderate/severe AATD with significantly reduced serum levels, but no genotyping was performed. Subsequent genotyping was not possible as the patients could not be contacted. Patients with alpha-1-antitrypsin deficiency were categorised into three groups based on serum levels: Reduced to normal (> 70 mg/dl and at least one deficiency allele), moderate (41–70 mg/dl) and severe (≤ 40 mg/dl) AATD group. As classification is predicated on serum levels and not genotype, certain genotypes, such as Pi SZ, are represented in different groups, see supplementary file 1.

### Pulmonary function

In all patients, body plethymography and a transfer factor measurement were performed in close temporal relation (< 30 days) to the CT examination date. Lung volumes were assessed by whole-body plethysmography and diffusing capacity for carbon monoxide (DLCO) by the single breath technique. Pulmonary function tests were performed according to the current guidelines of the American Thoracic Society and the European Respiratory Society [7–10]. In addition to forced expiratory volume in 1 s (FEV1), forced vital capacity (FVC) and its ratio FEV1/FVC, total lung capacity (TLC), functional residual capacity (FRC) and residual volume (RV) were measured.

### CT acquisition and reconstruction

Chest computed tomography was performed in all patients using a 64-channel multidetector CT scanner (Siemans Somatom Definition AS, Siemans Healthineers). Patients were placed in the supine position and CT scans were performed in the end-inspiratory breathing position to achieve near full lung capacity and avoid atelectasis. Scan parameters were 120 kVp, gantry rotation time 0.33 s (pitch 1.5), slice thickness 0.6 mm with 0.7 mm increments (reconstructed slice thickness 1 mm). In addition, each CT scan was visually analysed for significant motion artefacts by an experienced radiologist at the Thoraxklinik Heidelberg.

**Fig. 1:**
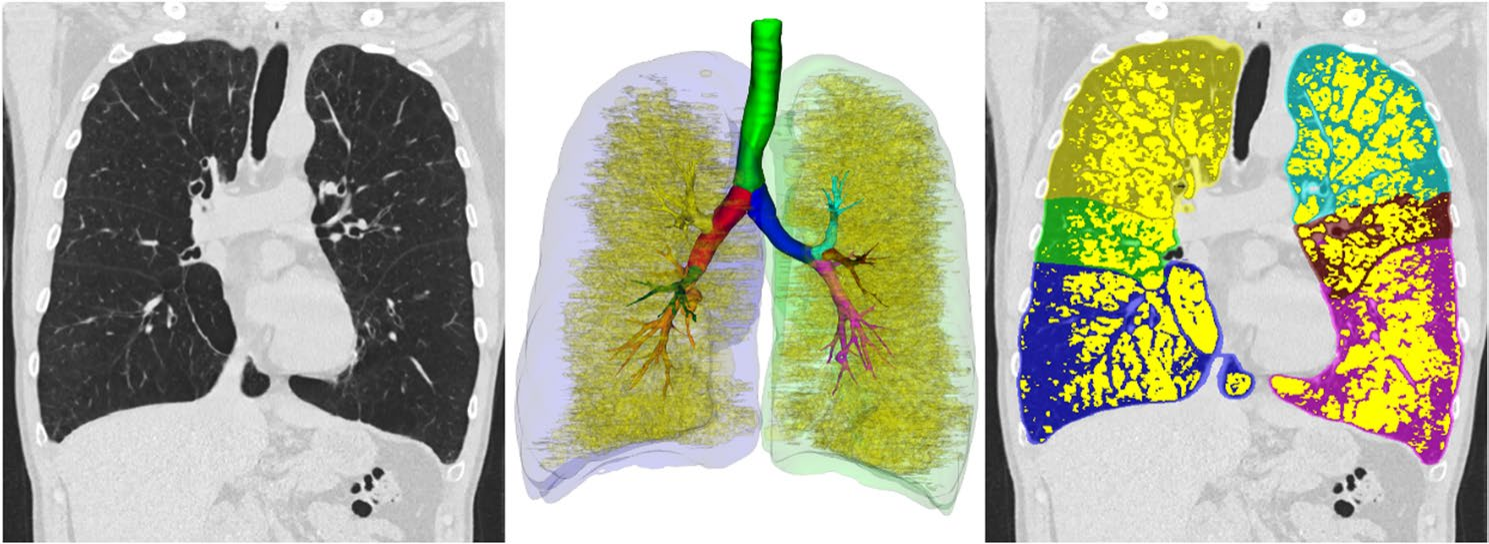
Segmentation of the lung using the YACTA software and quantification and characterisation of emphysema

**Fig. 2:**
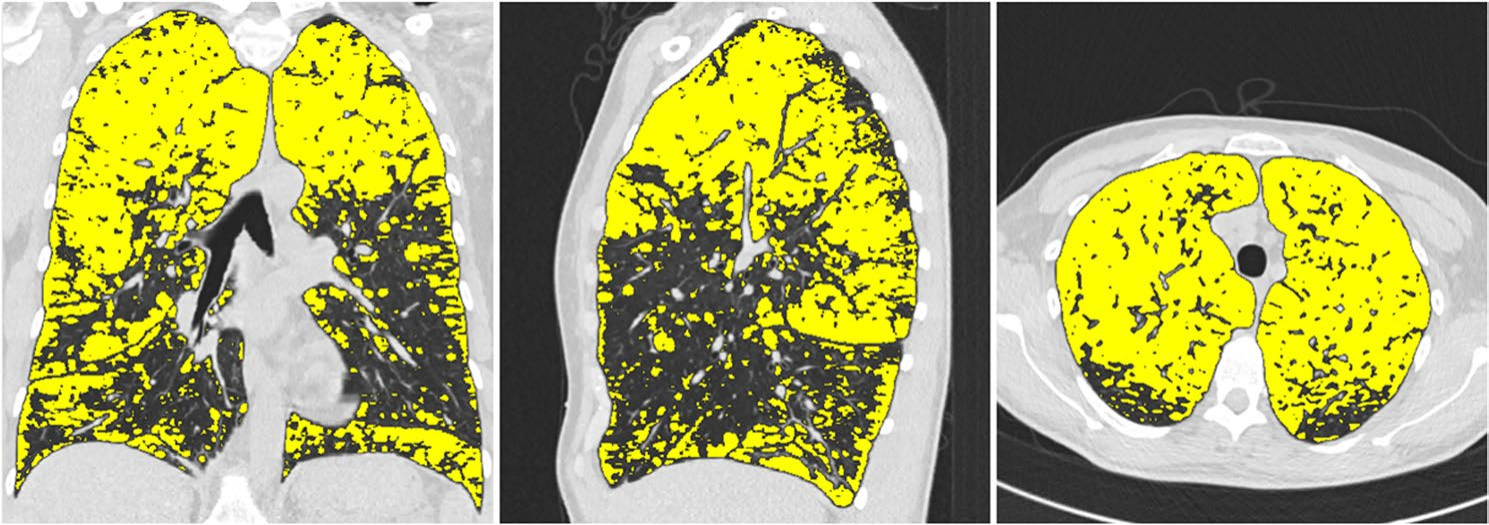
Illustration of typical upper lobe emphysema in a 50-year-old patient with MZ genotype and cumulative nicotine abuse of 3py. EI in %: Right upper lobe: 55, middle lobe: 37, right lower lobe: 32, left upper lobe: 55, lingula: 33, left lower lobe: 28

**Fig. 3:**
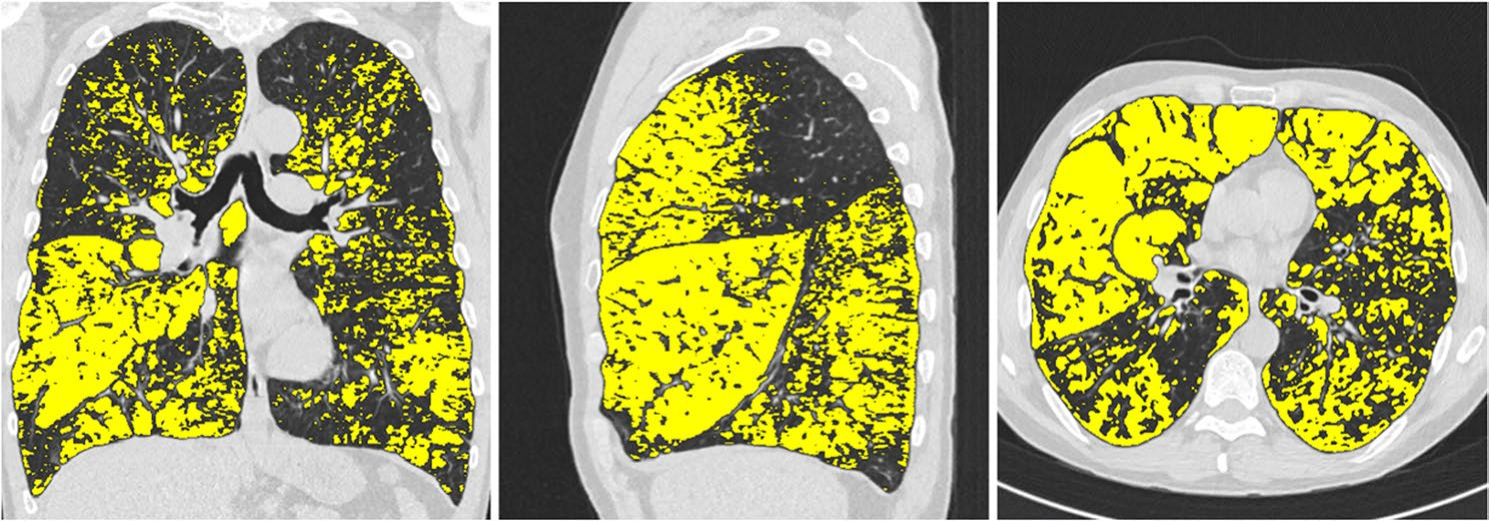
Illustration of typical upper lobe emphysema in a 57-year-old patient with ZZ genotype and cumulative nicotine abuse of 15py. EI in %: Right upper lobe: 38, middle lobe: 66, right lower lobe: 43, left upper lobe: 32, lingula: 51, left lower lobe: 38

The well-validated and fully automated YACTA software (Yet another CT analyser, version v2.9.4.98, programming by O.W.) segmented the airway tree and lung lobes on the inspiratory CT images. An analysis of the airways and lung parenchyma was carried out, various parameters describing e.g. pulmonary emhysema were determined, as previously described [3, 11–14]. The YACTA software not only offers comprehensive statistical analyses, but also a visualisation of the calculated emphysema area by colour overlaying the original CT images (see Figs. 1, 2 and 3).

The following parameters were included in the analysis: the mean lung density (MLD in HU), which describes the mean attenuation of all segmented lung voxels [15], the emphysema index (EI in %), which describes the ratio of emphysema voxels to all lung voxels, the 15th percentile density (PD-15), which is defined as the limit value in HU below which 15% o all voxels are distributed [16], the lung volume in cm³ of the segmented lung voxels. In addition, the position of the emphysematously affected pulmonary voxels was analysed. The following parameters were determined: ET1 =(|inner voxel|/|border voxel|) * 10. The greater this value, the larger the emphysema clusters; ET2 =(|peripheral voxel|/|emphysema voxel|)*100. The greater this value, the more predominant is the localization in the subpleural space; ET3 =(|panlobular voxel|/|emphysema voxel|) * 100. Similar to ET1, but this index provides first indications only if whole lobules are destroyed [17]. The above parameters were analysed for the entire lung and in the further analysis for each lobe (right upper lobe (RUL), middle lobe (RML), right lower lobe (RLL), left upper lobe (LUL), lingula (LLi), left lower lobe (LLL)).

In healthy lungs, the attenuation values are usually in the range of −750 to −850 Hounsfield Units (HU) with an average attenuation of 789 HU. In the context of pulmonary emphysema, the lung density is reduced due to the pathologically increased air accumulation. Threshold values below − 950 HU are often used to define pathological emphysema [2].

### Defining the lobar heterogeneity index

To distinguish between homogeneous and heterogeneous emphysema, the lung was divided into three distinct zones: the upper lobes, the middle lobe including the lingula, and the lower lobes. The emphysema index was calculated separately for each region. In instances where an absolute difference of more than 15% was identified between the lowest and highest emphysema indices, the emphysema was classified as heterogeneous [18]. Otherwise, the emphysema distribution was considered homogeneous. In patients with heterogeneous emphysema, the predominant emphysema type - upper lobe, middle lobe/lingula, or lower lobe - was determined by the region with the highest average emphysema index.

### Exclusion criteria

Exclusion criteria were previous lung surgery or lung volume-reducing interventions. Technical exclusion criteria were the administration of contrast medium, incorrect reconstruction of the knowledge-based and fully automated YACTA software, reconstructions other than I40f\3 and slice thicknesses of more than 1.25 mm [3].

### Statistical analysis

Statistical analyses were performed using the SPSS program, IBM SPSS Statistics (Version 29.0.0). Mean values, standard deviations (SD), absolute numbers and percentages (%) were used to describe the data. For the descriptive analyses, group comparisons were performed using Student’s t-test, the Man-Whitney U-test or the Chi-square test, as appropriate [19]. A stepwise binary logistic regression analysis was performed to identify independent predictors of emphysema predominantly affecting the middle lobe and lingula. The predictor variables - age, gender, serum AAT level, body mass index (BMI), packyears, FEV1 (% predicted) and RV - were entered into the model using a stepwise selection procedure based on statistical significance. Values of *p* < 0.05 were considered statistically significant.

## Results

### Patient characteristics

The study included a total of 75 patients. 13 patients (17.3%) had a reduced to normal AATD (88.5 ± 11.4 mg/dl), 16 patients (21.3%) had moderate AATD (57.9 ± 9.1 mg/dl) and 46 patients (61.3%) had severe AATD (16.7 ± 9.9 mg/dl). No statistically significant differences were observed between the three groups with regard to gender distribution, age and BMI.

The group of patients with reduced to normal AATD consisted of 8 patients (61.5%) with the genotype Pi MZ, 3 patients (23.1%) with the genotype Pi MS, 1 patient (7.1%) with the genotype Pi SZ and 1 patient (7.1%) with a rare variant (Pi MP lowell). In the group of patients with moderate AATD, 8 patients (50%) with the genotype Pi SZ, 4 patients (25.0%) with rare variants (Pi P-Lowell/Lowell, Pi ZP-Lowell, Pi SM procida, Pi ZI), 3 patients with the genotype Pi MZ (18.8%) and 1 patient (6.3%) with an unknown genotype were included. The group of patients with severe AATD comprised 36 patients (78.3%) with the genotype Pi ZZ, 7 patients (15.2%) with rare variants (Pi ZM procida, Pi ZM-Nichinan, Pi ZQ0HeidelbergI-IV, Pi ZVal79Glu.), 1 patient (2.2%) with the genotype Pi SZ and 2 patients (4.3%) with an unknown genotype. Genotyping was not performed in three patients and could not be performed retrospectively due to a lack of availability.

There were differences with regard to augmentation therapy and cumulative nicotine abuse. In the group with moderate AATD, 37.5% of patients received substitution therapy, whereas in the group with severe AATD, the figure was 69.6%. The group of patients with moderate AATD exhibited the highest levels of nicotine abuse (39.4 ± 31.9 py), followed by the group with severe AATD (15.1 ± 14.6 py) and the group with reduced to normal AATD (14.9 ± 17.2py). The analysis of smoking status revealed no statistically significant differences in the proportion of never, former and active smokers across the groups, see Table 1.

**Table 1:** Clinical characteristics of 75 patients who were included in the study

Variable	all *n* = 75	reduced to normal *n* = 13	moderate *n* = 16	severe *n* = 46	*P*
Sex (female)	38(50.7%)	3(23.1%)	9(56.3%)	26(56.5%)	0.091
Age (years)	54.3 ± 14.7	58.0 ± 16.2	54.7 ± 17.4	53.2 ± 13.3	0.574
BMI (kg/m²)	25.2 ± 5.9	27.4 ± 6.0	24.0 ± 5.8	24.9 ± 5.8	0.287
Serum level in mg/dl	38.0 ± 30.2	88.5 ± 11.4	57.9 ± 9.1	16.7 ± 9.9	**< 0.001**
*Genotyping*					
MS	3(4.0%)	3(23.1%)	0(0.0%)	0(0.0%)	**< 0.001**
MZ	11(14.7%)	8(61.5%)	3(18.8%)	0(0.0%)	**< 0.001**
SZ	10(13.3%)	1(7,7%)	8(50.0%)	1(2.2%)	**< 0.001**
ZZ	36(48.0%)	0(0.0%)	0(0.0%)	36(78.3%)	**< 0.001**
Rare variants	12(16.0%)	1(7.7%)	4(25.0%)	7(15.2%)	0.438
Unknown	3(4.0%)	0(0.0%)	1(6.3%)	2(4.3%)	0.681
Augmentation therapy	38(51.3%)	0(0.0%)	6(37.5%)	32(69.6%)	**< 0.001**
Smoking status					
Pack years	20.4 ± 22.1	14.9 ± 17.2	39.4 ± 31.9	15.1 ± 14.6	**< 0.001**
Never	19(25.3%)	4(30.8%)	4(25.0%)	11(23.9%)	0.881
Former	53(70.7%)	9(69.2%)	10(62.5%)	34(73.9%)	0.683
Active	3(4.0%)	0(0.0%)	2(12.5%)	1(2.2%)	0.139

*Abbreviations: AATD* Alpha-1-antitrypsin deficiency, *BMI* Body mass index, *N* number, *P* probabilit

### Lung function

The analysis of the patients revealed no statistically significant differences in terms of lung function parameters, GOLD classification and clinical categorisation, with the exception of FEV1 in L. The lowest observed FEV1 values were noted in patients diagnosed with moderate AATD. Conversely, patients with severe AATD exhibited the highest percentage of functional residual capacity (FRC) and, when considering the GOLD stages, there was considerable heterogeneity within the severe AATD group, which included patients with GOLD stages II-IV and those with no evidence of COPD. This heterogeneity was also reflected in the clinical categorisation. In the group with reduced to normal AATD, clinical group B (61.5%) dominated, and in the group with moderate AATD, group E (56.3%) dominated, see Table 2.

**Table 2:** Lung function and classification into GOLD groups and grades in the three groups studied

Variable	all *n* = 75	reduced to normal *n* = 13	moderate *n* = 16	severe *n* = 46	*P*
FEV_1_ (l)	1.8 ± 1.2	2.3 ± 1.3	1.3 ± 0.7	1.8 ± 1.2	**0.047**
FEV_1_ (% predicted)	54.1 ± 28.9	65.5 ± 30.4	42.0 ± 23.6	55.0 ± 29.1	0.084
FEV1/FVC	52.7 ± 18.9	61.3 ± 18.4	49.2 ± 17.8	51.5 ± 19.1	0.181
FRC (l)	5.0 ± 1.6	4.6 ± 1.2	4.9 ± 1.9	5.2 ± 1.7	0.481
FRC (% predicted)	159.6 ± 49.0	133.2 ± 35.5	153.6 ± 48.8	169.1 ± 50.2	0.055
RV (l)	4.0 ± 1.8	3.5 ± 1.1	4.1 ± 1.8	4.0 ± 1.9	0.586
RV (% predicted)	190.7 ± 80.4	155.5 ± 49.7	193.7 ± 68.7	199.7 ± 89.2	0.215
FRC/TLC	70.3 ± 12.3	65.6 ± 12.1	73.1 ± 12.7	70.6 ± 12.2	0.261
DLCO (% predicted) *	50.8 ± 22.9	59.2 ± 24.3	43.2 ± 19.7	51.1 ± 23.3	0.225
KCO (% predicted) *	64.3 ± 22.5	77.5 ± 25.3	61.1 ± 23.2	62.0 ± 20.7	0.104
GOLD grades					
GOLD I	1(1.3%)	1(7.7%)	0(0.0%)	0(0.0%)	0.089
GOLD II	18(24.0%)	2(15.4%)	2(12.5%)	14(30.4%)	0.255
GOLD III	16(21.3%)	3(23.1%)	4(25.0%)	9(19.6%)	0.888
GOLD IV	23(30.7%)	2(15.4%)	8(50.0%)	13(28.3%)	0.113
no COPD	17(22.7%)	5(38.5%)	2(12.5%)	10(21.7%)	0.245
Clinical categorisation (A/B/E) *					
Group A	18(24.0%)	3(23.1%)	3(18.8%)	12(26.1%)	0.836
Group B	29(38.7%)	8(61.5%)	4(25.0%)	17(37.0%)	0.123
Group E	28(37.3%)	2(15.4%)	9(56.3%)	17(37.0%)	0.077

*Abbreviations: AATD* Alpha-1-antitrypsin deficiency, *DLCO* Diffusion capacity for carbon monoxide, *FEV1* Forced expiratory volume in 1 second, *FEV1/FVC* Tiffeneau-index, *FRC* Functional residual capacity, *FVC* Forced vital capacity, *GOLD* Global Initiative for Chronic Obstructive Lung Disease, *KCO* DLCO/Va, *N* Number, *P* Probability

### Quantitative emphysema analysis

Quantitative computed tomography of the thorax (QCT) was used to analyse the lung parenchyma. In the present study, differences in the emphysema index and the different arrangements of the emphysema voxels (bullous, paraseptal, panlobular) were analysed.

Patients with severe AATD were found to have increased emphysema, which was reflected in a significant lower MLD (−819.8 ± 35.3 vs. −807.1 ± 55.2 vs. −774.0 ± 59.1, *p* = 0.007), a significant lower 15th percentile (−953.2 ± 32.0 vs. −942.5 ± 36.0 vs. −922.6 ± 58.1, *p* = 0.043) and a not significant higher EI (25.9 ± 17.9 vs. 20.2 ± 15.0 vs. 14.6 ± 17.5, *p* = 0.100), see Table 3. The lobe-specific analysis revealed that the EI in the group with severe AATD was most pronounced in the middle lobe, followed by the lingula and both lower lobe lakes, see Table 4.

**Table 3:** Lung parenchyma analysis using the YACTA software

Variable	all *n* = 75	reduced to normal *n* = 13	moderate *n* = 16	severe *n* = 46	*P*
MLD (HU)	−809.2 ± 47.2	−774.0 ± 59.1	−807.1 ± 55.2	−819.8 ± 35.3	**0.007**
EI (%)	22.7 ± 17.6	14.6 ± 17.5	20.2 ± 15.0	25.9 ± 17.9	0.100
LungVolume (cm³)	6853.8 ± 1796.8	6433.7 ± 1334.8	6419.3 ± 2099.6	7123.7 ± 1781.0	0.264
VesselVolume (cm³)	109.3 ± 30.7	121.4 ± 17.5	105.9 ± 43.1	107.1 ± 28.2	0.298
Perzentile 15 (HU)	−945.6 ± 39.5	−922.6 ± 58.1	−942.5 ± 36.0	−953.2 ± 32.0	**0.043**
ET1	21.7 ± 17.5	17.6 ± 18.0	15.8 ± 10.1	25.0 ± 18.8	0.123
ET2%	44.3 ± 9.8	45.3 ± 14.2	38.4 ± 9.8	46.0 ± 7.6	**0.025**
ET3%	16.4 ± 14.5	14.6 ± 16.2	10.5 ± 8.9	18.9 ± 15.1	0.120

*Abbreviations:* *AATD* alpha-1-antitrypsin deficiency, *EI* Emphysema index, *ET1* (|Inner voxel| / |border voxel|) * 10, *ET2* (|Peripheral voxel| / |emphysema voxel|)*100, *ET3* (|Panlobular voxel| / |emphysema voxel|) * 100, *HU* Hounsfield unit, *MLD* Mean lung density, *PD15* 15th percentile density of the lung, *N* Number, *P* Probability

**Table 4:** lobe-specific analysis of the emphysema index using the YACTA software and analysis of the distribution of pulmonary emphysema

Emphysema-Index	all *n* = 75	reduced to normal *n* = 13			moderate *n* = 16		severe *n* = 46		*P*
Right lung	22.5 ± 18.1	13.9 ± 18.5			19.7 ± 15.0		25.8 ± 18.3		0.086
Right upper lobe	19.3 ± 18.5	17.0 ± 23.8			21.4 ± 18.9		19.3 ± 17.1		0.821
Middle lobe	28.2 ± 22.5	10.7 ± 12.8			21.4 ± 15.6		35.5 ± 23.4		**< 0.001**
Right lower lobe	21.7 ± 20.3	8.9 ± 15.1			17.3 ± 15.2		26.9 ± 21.5		**0.010**
Left Lung	22.9 ± 17.6	15.3 ± 16.8			20.3 ± 16.0		25.9 ± 17.9		0.126
Left upper lobe	19.2 ± 17.4	17.3 ± 21.1			22.1 ± 18.7		18.8 ± 16.2		0.740
Lingula	25.5 ± 20.2	12.7 ± 13.1			21.6 ± 19.2		30.5 ± 20.6		**0.011**
Left lower lobe	22.4 ± 20.9	11.0 ± 15.5			16.6 ± 15.8		27.6 ± 22.3		**0.017**
*homgeneous and heterogeneous emphysema*									
homogeneous	38(50.7%)	9(69.2%)			11(68.8%)		11(68.8%)		**0.042**
heterogeneous									
upper lobe predominant	8(10.7%)	3(23.1%)			4(25.0%)		1(2.2%)		**0.011**
middle lobe and lingula predominan	22(29.3%)	1(7.7%)			0(0.0%)		21(45.7%)		**<0.001**
lower lobe predominant	7(9.3%)	0(0.0%)			1(6.3%)		6(13.0%)		0.322

*Abbreviations:* *AATD* Alpha-1-antitrypsin deficiency, *N* Number, *P* Probability

**Fig. 4:**
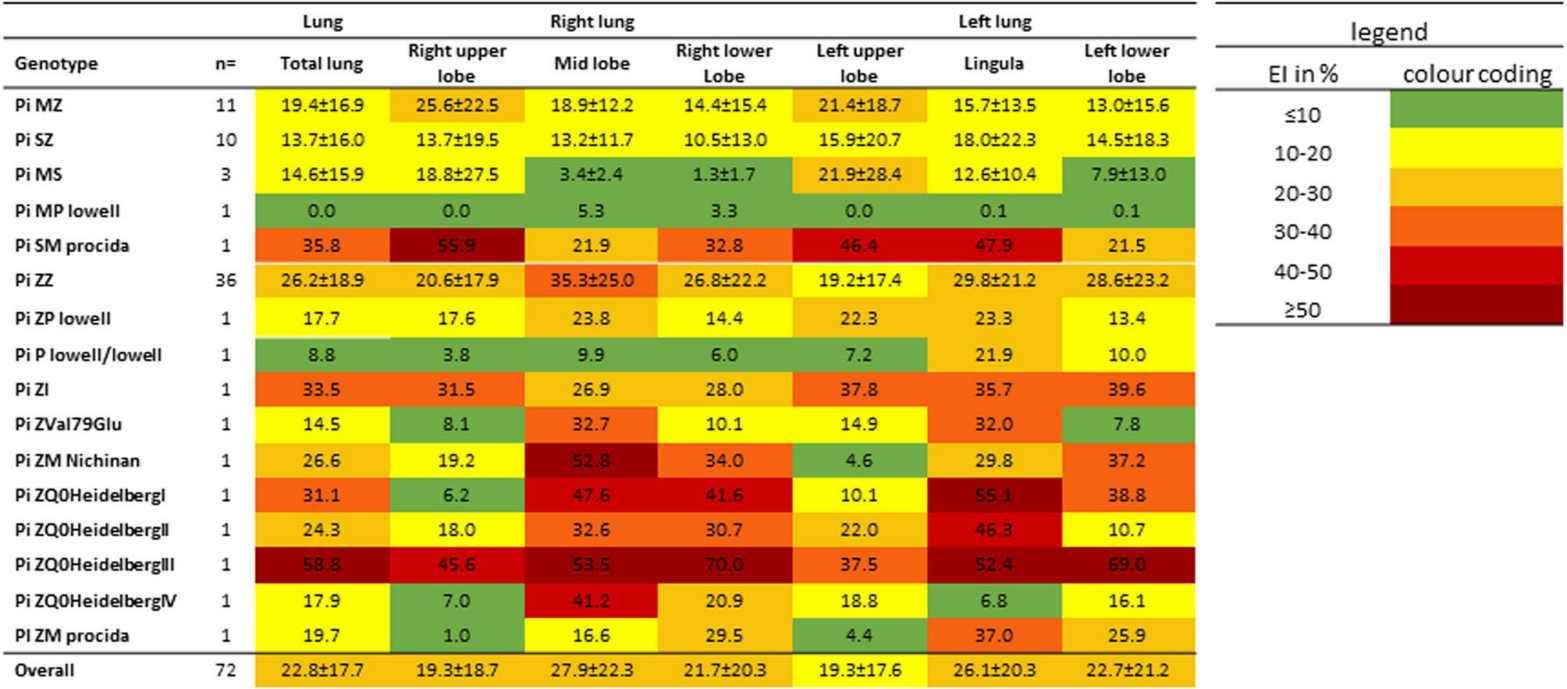
Heatmap of the association between genotypes in mild and severe AATD and the lobe-specific index. The severity of the emphysema index is marked in color (from green, ≤ 10, to dark red, ≥ 50)

Figure 4 shows the expression of the lobe-specific emphysema index for the different genotypes in the form of a heat map. Patients with a Pi MZ genotype had predominantly affected upper lobes. Pi SMprocida has the strongest expression of EI in cases with moderate AATD, which is similar to that in patients with severe AATD. In severe AATD cases, the middle lobe and lingula were most affected in some patients (Pi ZVal79Glu, Pi ZQ0HeidelbergI, Pi ZQ0HeidelbergII), while the lower lobes were most affected in Pi ZQ0HeidelbergIII. Furthermore, the type of emphysema was analysed with regard to bullous, paraseptal and panlobular components. A significant group difference was only found in the paraseptal components (ET2), whereby the group with reduced to normal and the group with severe AATD did not differ significantly from the mean value. In contrast, no group differences could be determined for the bullous (ET1) and panlobular parts (ET3), see Table 3.

According to the above definition, 38 patients (50.7%) presented with homogeneous emphysema, 22 patients (29.3%) with middle- or lingular-lobe-predominant emphysema, eight patients (10.7%) with upper-lobe-predominant emphysema, and seven patients (9.3%) with lower-lobe-predominant emphysema. Homogeneous and upper-lobe-predominant emphysema were most commonly observed in the groups with reduced to normal and moderate AATD, whereas middle- and lingula-predominant heterogeneous emphysema were significantly more prevalent among patients with severe AATD. There were no differences between the three groups in terms of lower lobe predominant emphysema, see Table 4.

Based on these findings, a stepwise binary logistic regression was performed with emphysema predominantly affecting the middle lobe and lingula as the outcome variable. The initial model included the following covariates: age, gender, serum AAT level, BMI, pack years, FEV1 (% predicted) and residual volume (RV). Of these, only age (CI 1.01–1.12, *p* = 0,024) and serum AAT level (CI 0.92–0.98, p = < 0.001) emerged as relevant independent factors associated with the outcome. Consequently, age and serum AAT level were retained in the final model, together with gender and BMI, which were not significantly associated with the outcome, but were included for adjustment purposes, see Table 5.

**Table 5:** Cox regression analysis to assess the independent risk factors associated with emphysema predominantly affecting the middle lobe and lingula

Variable	B	HR	95% CI of coefficient B lower upper		*P*
Age	0.06	1.07	1.01	1.12	**0.024**
Gender	0.40	1.49	0.43	5.10	0.527
AAT Serum level	−0.05	0.95	0.92	0.98	**< 0.001**
BMI	−0.07	0.93	0.84	1.03	0.179

The table presents the results of the binary logistic regression analysis with emphysema predominantly affecting the middle lobe and lingula as the outcome variable

*AAT* Alpha-1-antitrypsin, *B* Regression coefficient, *BMI* Body mass index, *CI* Confidence interval, *HR* Hazard ratio, *P* Probability, *N* = 75, Statistically significant p-values (p < 0.05) are shown in bold.

## Discussion

The aim of our analysis was to investigate emphysema in patients with different AATD genotypes using quantitative computed tomography. The study of patients with reduced to normal, moderate and severe AATD shows relevant differences between these three groups.

Although no significant differences in lung function (FEV1%) were observed between the groups, significant differences were found in the QCT analyses. A decrease in mean lung density and 15th percentile of lung density were noted in patients with lower serum levels (*p* < 0.05), with no differences observed in the global emphysema index. Lobe-specific analysis revealed that the reduced-to-normal and moderate AATD groups had upper lobe-predominant emphysema, while the severe AATD group had basal-predominant emphysema, with the most severe damage observed in the middle lobe and lingula. Logistic regression with emphysema predominantly affecting the middle lobe and lingula as the outcome variable revealed that lower serum levels of AAT and higher age were significant independent predictors of this emphysema pattern. It is plausible that age is an independent predictor, as patients must reach a certain age before developing emphysema. These findings provide new insights into the distribution of emphysema in AATD and suggest that in severe cases, the disease may not predominantly affect the lower lobes, as previously thought.

The congruent and significant differences in a number of different emphysema parameters we analysed, such as MLD and PD15 reinforce the results and suggest an additional diagnostic value of QCT in AATD. Additionally, the middle lobe is known to be more vulnerable to infections, as evidenced by an increased incidence of NTM infections, particularly in patients with bronchiectasi [20]. Infections play a crucial pathophysiological role in AATD, as they lead to the recruitment of granulocytes, which subsequently result in the local secretion of neutrophil elastase, exacerbating tissue damage. It is plausible that the pronounced emphysema involvement in the middle lobe in patients with severe AATD may be linked to an increased susceptibility of this lobe to infections. Moreover his finding aligns with the literature, which describes the middle lobe as a common target for endoscopic lung volume reduction in patients with AATD [21].

Data are already available on the relationship between quantitative chest computed tomography and lung function. In general, it is known that the development of emphysema precedes the decline in lung function by years [22]. A number of studies have demonstrated that mild to moderate emphysema can be present in the absence of a reduction in FEV1 [23, 24]. However, there is a correlation between diffusion capacity and the pathological extent of emphysema, although diffusion capacity shows relatively high variability and is not specific for emphysema [25]. In addition, quantitative computed tomography with measurement of the emphysema index was found to have a stronger correlation with mortality than FEV1 [6]. As demonstrated in the study by Dawkins et al., the CT emphysema index in the upper lung zones exhibited the strongest correlation with mortality, while lung function demonstrated no independent effect on survival [6]. It has been established that patients suffering from alpha-1-antitrypsin deficiency (AATD) are more prone to the development of basal emphysema. In addition, approximately one third of patients have been diagnosed with apical emphysema [26]. Theories concerning the increased loss of forced expiratory volume in basally emphasised emphysema hypothesise that the augmented volume in the lower zones of the lungs results in more intensive functional impairment [27]. It is also hypothesised that the airways in the lower lung regions undergo greater collapse during expiration due to a deficiency in supporting tissue [28]. Comparative studies between patients with AATD and COPD patients without AATD demonstrated that emphysema was more severe in AATD patients, despite similar lung function parameters being found in both groups [29].

The studies cited so far have analysed the basal and apical parts of the lung for the extent of emphysema; the middle part of the lung was often not included. In addition, older studies used a purely visual analysis rather than a quantitative analysis of emphysema. This study is a novelty in that all lung sections, especially the middle lobe and the lingula, were analysed on a lobe-specific basis. The results challenge the paradigm of basal emphysema in patients with AATD.

AATD is a rare disease, with only about one per cent of all screening tests performed in Germany revealing a rare mutation [30]. Therefore, the inclusion of rare mutations such as Pi QOHeidelbergI-IV is a strength of the study. Previous studies have mainly considered patients with the genotypes Pi SZ and Pi ZZ. Looking at the expression of the emphysema index in the rare mutations, a heterogeneous picture emerges. In some cases, the middle lobes and the lingula were most severely affected (Pi ZVal79Glu, Pi ZQ0HeidelbergI, Pi ZQ0HeidelbergII), while in others the lower lobes were most severely affected (Pi ZQ0HeidelbergIII). The highest emphysema index (EI) was observed in the rare mutation Pi ZQ0HeidelbergIII. This observation can be explained, among other things, by the pronounced nicotine abuse of 40 py cumulatively. In this context, it should be mentioned that studies suggest that there is a non-linear relationship between pack-years and FEV1 loss [31]. Castaldi et al. were able to demonstrate that there is a plateau phase in which additional pack-years are not associated with a further decline in FEV1. The levelling of the FEV1/pack-year relationship occurred in COPD patients without AATD at a smoking exposure of 40–60 pack-years and in patients with AATD at around 20 pack-years [31]. With a cumulative nicotine abuse of 25 py, Pi Q0HeidelbergIV showed a whole lung emphysema index comparable to that of patients with mild AATD. The lobe-specific analysis, however, shows emphysema with a clear focus on the middle lobe.

There is currently insufficient knowledge to predict the clinical course of rare mutations. The heterogeneity of severity and location of emphysema in rare mutations leads to the conclusion that serum levels alone cannot be used as a prognostic tool for the risk of developing emphysema. In order to determine the disease status and the optimal time to start augmentation therapy in patients with rare mutations, a holistic view should be taken, including not only protein structure but also imaging, comorbidities, lung function and laboratory parameters. In this context, QCT is proving to be an essential tool for the detection of emphysema. In the future, an increase in the detection of rare mutations can be expected due to the increasing number of screening tests. Further prospective studies could contribute to a better understanding of the disease progression of the different rare mutations.

### Limitations

As this was a retrospective study, only data already collected in daily clinical practice could be used for analysis. The lung index status was not recorded for each patient in the study, which is why no further analysis could be carried out in this regard. Due to the monocentric nature of the study, it is important to note the sample size, which could be increased by a multicentre approach. In addition, lung CT scans were performed on the basis of different indications (clinical symptoms, assessment of lung status with positive family history). It should also be noted that 3 patients were found with significantly reduced serum levels, but no genotyping was performed. Subsequent genotyping was not possible because the patients could not be contacted. As AATD is a heterogeneous disease, the results should ideally be evaluated in a larger cohort such as the international AATD registry. At the Thoraxklinik, the cohort comprises both patients with mild disease courses, who presented for consultation or inclusion in the EARCO registry following diagnosis, as well as individuals with severe COPD. The latter group includes patients seeking valve therapy as well as those referred for other reasons, such as follow-up care or registry inclusion. Consequently, it is not possible to completely eliminate the possibility of a selection bias in the sample.

## Conclusion

The study highlights that the severity and pattern of emphysema in individuals with Alpha-1 Antitrypsin Deficiency (AATD) are influenced by both the degree of AAT deficiency and the specific genetic variants they carry. It finds that patients with severe AATD experience more widespread and differently distributed emphysema compared to those with milder forms of the condition, even when lung function measurements are similar. Contrary to the commonly held belief that the lower lobes are most affected in severe AATD, the study reveals that the middle lobe is actually the most affected region. The findings underscore the added value of imaging techniques in evaluating AATD, which provide more detailed insights than lung function tests alone. The study also stresses the importance of tailored diagnosis and phenotyping, especially for patients with rare allele variants of AATD.

The table presents the results of the binary logistic regression analysis with emphysema predominantly affecting the middle lobe and lingula as the outcome variable. AAT = alpha-1-antitrypsin; B = regression coefficient. BMI = body mass index. CI: confidence interval. HR = hazard ratio. P = probability. *N* = 75.

## Data Availability

The full data set supporting the conclusions of this article is available upon request from FCT.

## Abbreviations

AAT: Alpha-1-Antitrypsin
AATD: Alpha-1-Antitrypsin Deficiency
BMI: Body Mass Index
COPD: Chronic obstructive pulmonary disease
CT: Computed tomography
DLCO: Diffusion capacity for carbon monoxide
EI: Emphysema index
ET1: ET1 =(|inner voxel|/|border voxel|) * 10
ET2: ET2 =(|peripheral voxel|/|emphysema voxel|)*100
ET3: ET3 =(|panlobular voxel|/|emphysema voxel|) * 100
FEV1: Forced expiratory volume in 1 s
FEV1/FVC: Tiffeneau-index
FRC: Functional residual capacity
FVC: Forced vital capacity
GOLD: Global Initiative for Chronic Obstructive Lung Disease
HU: Hounsfield unit
IEF: Isoelectric focusing
MLD: Mean lung density
PD15: 15th percentile density of the lung
Pi: Proteinase Inhibitor
py: Pack year
QCT: Quantitative computed tomography
RV: Residual volume
SD: Standard deviation
TLC: Total lung capacity
YACTA: Yet another CT analyzer
